# Disease-specific out-of-pocket healthcare expenditure in urban Bangladesh: A Bayesian analysis

**DOI:** 10.1371/journal.pone.0227565

**Published:** 2020-01-14

**Authors:** Md. Mahfuzur Rahman, Cherri Zhang, Khin Thet Swe, Md. Shafiur Rahman, Md. Rashedul Islam, Md. Kamrujjaman, Papia Sultana, Md. Zakiul Hassan, Md. Shahinul Alam, Md. Mizanur Rahman

**Affiliations:** 1 Global Public Health Research Foundation, Dhaka, Bangladesh; 2 Department of Global Health Policy, The University of Tokyo, Tokyo, Japan; 3 Department of Mathematics, University of Dhaka, Dhaka, Bangladesh; 4 Department of Statistics, University of Rajshahi, Rajshahi, Bangladesh; 5 icddr,b, (Formerly, International Centre for Diarrheal Disease Research Bangladesh) Dhaka, Bangladesh; 6 Bangabandhu Sheikh Mujib Medical University, Dhaka, Bangladesh; Institute of Economic Growth, INDIA

## Abstract

**Background:**

Because of the rapid increase of non-communicable diseases (NCDs) and high burden of healthcare-related financial issues in Bangladesh, there is a concern that out-of-pocket (OOP) payments related to illnesses may become a major burden on household. It is crucial to understand what are the major illnesses responsible for high OPP at the household level to help policymakers prioritize key areas of actions to protect the household from 100% financial hardship for seeking health care as part of universal health coverage.

**Objectives:**

We first estimated the costs of illnesses among a population in urban Bangladesh, and then assessed the household financial burden associated with these illnesses.

**Method:**

A cross-sectional survey of 1593 randomly selected households was carried out in Bangladesh (urban area of Rajshahi city), in 2011. Catastrophic expenditure was estimated at 40% threshold of household capacity to pay. We employed the Bayesian two-stage hurdle model and Bayesian logistic regression model to estimate age-adjusted average cost and the incidence of household financial catastrophe for each illness, respectively.

**Results:**

Overall, approximately 45% of the population of Bangladesh had at least one episode of illness. The age-sex-adjusted average medical expenses and catastrophic health care expenditure among the households were TK 621 and 8%, respectively. Households spent the highest amount of money 7676.9 on paralysis followed by liver disease (TK 2695.4), injury (TK 2440.0), mental disease (TK 2258.0), and tumor (TK 2231.2). These diseases were also responsible for higher incidence of financial catastrophe. Our study showed that 24% of individuals who suffered typhoid incurred catastrophic expenditure followed by liver disease (12.3%), tumor (12.1%), heart disease (8.4%), injury (7.9%), mental disease (7.9%), cataract (7.1%), and paralysis (6.5%).

**Conclusion:**

The study findings suggest that chronic illnesses were responsible for high costs and high catastrophic expenditures in Bangladesh. Effective risk pooling mechanism might reduce household financial burden related to illnesses. Chronic illness related to NCDs is the major cause of OOP. It is also important to consider prioritizing vulnerable population by subsidizing the high health care cost for some of the chronic illnesses.

## Introduction

The double burden of disease poses a major challenge for low- and middle-income countries (LMICs) with fragile health systems [1–4]. In low-and-middle-income countries, public funding for health services are insufficient and risk-pooling mechanisms is limited or unavailable. Non-communicable diseases (NCDs) and infectious diseases cause financial catastrophe directly by means of out-of-pocket (OOP) spending on treatment, and indirectly by limiting labor participation in income-generating activities [5–8]. Chronic diseases and its related comorbidities have recently become potential health agenda even in high-income countries (HICs). In HICs, existing health insurance policies are struggling to cope up with the treatment cost of chronic illnesses. Even Japan is currently planning to reform its health insurance system according to disease burdens and population structure. Therefore, assessment on the cost of illness and its financial impact on households may reveal opportunities for a country’s policy planners to start or stabilize universal health coverage (UHC).

In the Asia-Pacific region, Bangladesh encounters the highest rate of catastrophic expenditure (17%), and about 24% of the poorest households and 7% of the richest households are forced to borrow money or sell household assets to pay for costs associated with illnesses [9]. Despite these alarming statuses, health insurance in Bangladesh is almost nonexistent except for a few very small pockets of NGO-sponsored plans [10]. In 2030, 20% of the population will encounter catastrophic health expenditure and about 10% of those will be pushed into poverty for seeking health care unless health care policy changes [11]. Neighboring country, Nepal, has been implementing a subsidy program for underprivileged population suffering from cancer and renal diseases. Even with the existence of subsidy program in Nepal, 40% of the people who suffered from kidney and liver diseases and cancer still encountered catastrophic health expenditure [12].

Despite the rising epidemic of NCDs and high burden of OOP health care payment, there is no subsidy program for vulnerable population who suffer from chronic illnesses in Bangladesh. Only a few studies explored the financial burden of some specific acute illnesses such as diarrhea and influenza [13, 14]. None of the study in Bangladesh has estimated how much households spent on a comprehensive list of illnesses when they received treatment and which illnesses pushed households into financial catastrophe. Information on medical expenses for each disease is not only essential for fixing costs at the health facility level but also critical for launching health insurance scheme. It is important for Bangladesh to explore the illness specific health care cost and its financial burden on household as the country is trying to achieve UHC. This will provide critical evidence to the government and policy makers to implement effective interventions to protect people from financial catastrophe. Our study has successfully solved the comorbidity cost issues through complex analysis. First, we reported the prevalence of all illnesses in the previous 30 days, and then estimated the average medical expense for each reported illness separately by using a Bayesian two-stage hurdle model. We also examined the adjusted and unadjusted incidence of financial catastrophe for each illness using Bayesian logistic regression models with non-informative prior information.

## Data and methods

### Study design

This study was done in Rajshahi city in the northwest region of Bangladesh. It is the third largest city in the country and broadly represents many urban areas in Bangladesh. The average household size in Rajshahi district is around five and its population is more than two million. This study was cross-sectional in nature, based on a three-stage, cluster-sampling methodology. We collected information on 1600 households from August to November 2011. The details of the research protocol and study design are described elsewhere. The overall response rate in our study was more than 99%.

### Data collection

We recruited 27 interviewers (social scientist, demographers and graduates of statistics with experience in survey methods) and five supervisors to administer the survey. All of them received 10 days of training and two days of practical sessions on the content of the questionnaires, techniques to elicit more information, and strategies to obtain complete and reliable data. The respondents in this study were either the female or the head of the household, and occasionally, the person with the most knowledge. We acquired informed consent prior to conducting the interviews. Interviewers recorded information by face-to-face direct interview method using a pretested, structured questionnaire. Questions included household member’s socio-demographic characteristics, household consumption expenditure, and all types of illnesses that suffered in the previous 30 days. In the structured questionnaire, a disease list guided the illness coding which was developed based on previous studies, [2, 6, 15–17] and then finalized after pilot testing among 100 households. Data were collected separately for each illness based onset or duration, diagnosis, treatment response, treatment cost and coping strategies, and care-seeking behavior level (n = 4461) which are nested within household (n = 1593). Out of the 4461 episodes of illnesses, 4127 episodes incurred positive expenditure and the rest of diseases did not require any spending i.e. zero expenditure. Regarding the information on health care expenditure, subjects were asked about the amount they paid for the care they received for each illness episode in the past 30 days. In the case of comorbidity or joint cost, we put additional codes during data collection time for future analysis.

### Allocation of comorbidity costs

Costs for comorbidities are common phenomenon in illness-related healthcare expenditure survey. Because of the same doctors writing the same prescriptions, it is difficult for patients to differentiate cost of diabetes versus hypertension, or hypertension versus heart diseases, and so on. In this study, around 14% of the outpatient costs were jointly reported with other illnesses. Although several methods exist to allocate comorbidity costs, we used random effects regression models with multiple imputations for this study. Details of the methodological issues are described in a previous paper [18]. The primary outcome variable was OOP payments in the cost allocation process, and the predictor variables were age, gender, care-seeking behavior, number of illnesses, and the presence of chronic illnesses in the household.

### Outcome variables

The primary outcome was total OOP medical expenditures for each episode of illness in the past 30 days prior to survey. This measure includes all health care expenditures for inpatient, outpatient, traditional healers, self-medication, home care, and medical supplies and equipment.

### Statistical analysis

We estimated summary statistics and frequency distributions for some selected variables. Due to the skewed and zero-truncated observations of the outcome, we used a Bayesian two-stage hurdle model for estimating unadjusted and age-sex-adjusted average medical expenditure for each episode of illness. Bayesian two-stage hurdle model allows us to consider zero cost expenditure. Bayesian method addresses issues related to under reported illnesses due to the nature of small sample size in some chronic illnesses. The first stage hurdle involves the decision whether or not to participate in health care expenditure due to illness and was modeled with logistic regression to include zero cost illnesses. The second hurdle addresses the level of health care expenditure and models the log-transformed positive costs with linear regression. Finally, the two models were combined, with the probability of incurring a health care cost multiplied by expected cost to get average health expenditure for specific illnesses.

Consistent with previous studies, catastrophic spending of illness was defined when health expenses exceeded 40% threshold of household capacity to pay [19–21].The household capacity to pay is defined as a household non-subsistence spending after satisfying the subsistence needs (food expenditure). The incidence of catastrophic expenditure may be rare for some specific illness, so we employed Bayesian logistic regression model to estimate unadjusted and multivariable-adjusted model (age, sex and total household consumption). All analyses at both the univariate and multiple regression stages were adjusted for probability sample design. Data analyses were performed using JAGS and R.

### Ethical considerations

This study received ethical approval from the Research Ethics Committee of the University of Tokyo and the Bangladesh National Research Ethics Committee with reference number BMRC/NREC/2010-2013/1161. The consent form, questionnaire, self-reported illness, and disease codes were approved by the Ethics Committee together with the study protocol. This consent form contained information on the objectives of the study, risks, benefits and freedom of participation, and confidentiality.

## Results

### Background characteristics and prevalence of morbidity

Table 1 presents the key characteristics of the surveyed households and their members. The average household size in urban area was 4.6 (95% CI: 4.5–4.7).The most commonly reported illnesses among study populations were NCDs such as hypertension (7.2%), rheumatic arthritis (5.1%), heart diseases (2.8%), diabetes (3.8%), gastritis/peptic ulcer (5.4%), and asthma (2.0%), whereas infectious diseases such as cold/fever (18.4%), and diarrhea/gastroenteritis (2.0%) were also featured among the top 20 illnesses (S1 Table).

**Table 1:** Descriptive statistics of survey households, household members and treatment behavior, Bangladesh, 2011.

Characteristic	No.	Percent (95% CI)
Household size (no. of members)		
1–2	127	7.6 (6.3–9.2)
3–5	1132	69.7 (67.2–72.2)
≥ 6	334	22.7 (20.3–27.9)
Gender of household member		
Male	3590	49.9 (48.7–51.1)
Female	3612	50.1 (48.9–51.4)
Age of household members (years)		
0–4	449	6.2 (5.7–6.9)
5–9	565	7.8 (7.1–8.6)
10–14	740	10.6 (9.7–11.5)
15–29	2128	29.8 (28.3–31.4)
30–44	1612	22.4 (21.3–23.5)
45–59	1119	15.3 (14.3–16.3)
≥60	589	7.9 (7.2–8.7)
Education status of household members		
No education	1265	18.0 (16.2–19.9)
Primary	1831	26.2 (23.7–28.9)
Secondary	2002	28.3 (27.0–29.7)
Higher education	2104	27.5 (24.1–31.2)
Care-seeking behaviour		
Inpatient	72	1.7 (1.3–2.3)
Outpatient		
At public facility only	622	14.2 (12.0–16.8)
At private facility only	904	19.0 (16.3–22.2)
At both public and private facilities	87	1.9 (16.3–22.2)
Traditional healer	221	5.0 (3.9–6.4)
Self-medication/no treatment sought	2555	58.2 (54.1–62.2)

CI, confidence interval.

### Average cost of illness

Table 2 presents the results of Bayesian two-stage hurdle modeling of the average medical expenditure among individuals who reported illness in the past 30 days prior to survey. Household spent anywhere from TK 1000 to TK 7676 for treating chronic diseases including kidney stone (TK 1409.7), cataract (TK 1559.7), heart (TK 1954.0), urinary tract infection (TK 2060.8), tumor (TK 2231.2), mental disease (TK 2258.0), injury (2440.0),liver disease (TK 2695.4), paralysis (TK 7676.9) and so on. Household spent TK 500 to TK 1000 for asthma, rheumatic arthritis, diabetes, dental, and pneumonia. Typhoid fever caused households to spend the highest health care cost (TK 1814.0) among acute illnesses. Healthcare expenditure was less than TK 500 for treating tropical illnesses and symptomatic illnesses such as cold/fever, hypertension, diarrhea/gastroenteritis, allergy, gastritis, migraine/headache, otitis media, hemorrhoids, physical weakness, insomnia and skin disease.

**Table 2:** Unadjusted and multivariable adjusted average medical expenditure of reported illness, Bangladesh, 2011.

Illness	Mean costs (95% Crl)		
	Unadjusted		Multivariable adjusted
Cold/fever	183.7 (179.9–188.2)		170.9 (167.0–176.0)
Hypertension	493.2 (477.4–511.8)		509.8 (475.5–555.5)
Gastritis/Peptic ulcer	286.0 (278.4–296.3)		280.0 (266.7–300.8)
Rheumatic arthritis	566.7 (532.6–613.1)		467.9 (433.8–511.4)
Diabetes	785.2 (746.9–830.8)		794.7 (749.8–853.7)
Heart diseases	1954.0 (1840.6–2135.4)		1984.0 (1771.0–2361.0)
Migraine/Headache	307.4 (280.3–361.4)		275.6 (242.8–335.5)
Asthma	553.0 (489.8–661.8)		557.0 (445.7–739.0)
Diarrhea/Gastroenteritis	493.4 (465.0–537.9)		500.6 (444.6–594.9)
Allergy	312.6 (274.2–391.4)		273.3 (228.2–360.1)
Injury	2440.0 (2167.6–2824.4)		1702.3 (1274.0–2653.0)
Skin disease	475.4 (404.1–614.9)		441.9 (353.6–599.5)
Cataract	1559.7 (1245.2–2332)		1030.3 (666.9–1865.6)
Dental	715.1 (622.0–885.9)		885.0 (597.9–1519.8)
Kidney stone	1409.7 (987.7–2281.0)		1634.4 (896.6–3654.6)
Haemorrhoids	455.6 (366.0–605.4)		377.8 (274.9–615.6)
Urinary tract infection	2060.8 (1757.2–2711.0)		3034.9 (1828.0–6613.0)
Liver disease	2695.4 (2028.4–4737.0)		3165.6 (1659.0–8323.0)
Otitis media	415.9 (323.2–542.8)		595.0 (323.9–1668.3)
Tumour	2231.2 (1482.1–4130.7)		20096.0 (1156.0–50123.0)
Typhoid	1814.0 (1814.0–1814.0)		2104.4 (1786.0–3045.0)
Mental disease	2258.0 (1883.4–2487.0)		2853.7 (1838.0–6431.0)
Physical weakness	278.0 (227.7–326.6)		272.6 (212.7–399.0)
Pneumonia	585.9 (585.9–585.9)		761.1 (587.0–1445.4)
Paralysis	7676.9 (405.3–4174.4)		NA
Insomnia	265.9 (201.9–398.5)		1103.3 (206.8–1438.2)

CrI, credible interval.

### Economic burden of illness

Table 3 presents the results of Bayesian regression modeling of the incidence of catastrophic expenditure for each illness episode. Again, majority of the chronic diseases were responsible for higher incidence of catastrophic expenditure. The highest percent (24.2%) of catastrophic expenditure households faced was to pay for typhoid illness, followed by liver diseases (12.3%), tumor (12.1%), heart disease (8.4%), injury (7.9%), mental disease (7.9%) and cataract (7.1%). Kidney stone (6.2%) and paralysis (6.5%) were also big contributors to catastrophic expenditure.

**Table 3:** Incidence of catastrophic health expenditure of top illnesses.

Illness	Incidence of catastrophic payments (95% Crl)	
	Unadjusted	Multivariable adjusted
Cold/fever	1.8 (1.2–2.6)	1.8 (1.2–2.6)
Hypertension	1.3 (0.5–2.4)	1.3 (0.5–2.4)
Gastritis/Peptic ulcer	1.0 (0.3–2.3)	1.0 (0.3–2.2)
Rheumatic arthritis	3.3 (1.7–5.3)	3.3 (1.7–5.3)
Diabetes	1.4 (0.4–3.0)	1.4 (0.4–3.0)
Heart diseases	8.4 (5.2–12.5)	8.4 (5.1–12.4)
Migraine/Headache	0.6 (0.0–2.0)	0.6 (0.0–2.0)
Asthma	3.3 (1.1–6.7)	3.3 (1.2–6.3)
Diarrhea/Gastroenteritis	4.3 (1.6–8.2)	4.3 (2.0–7.3)
Allergy	1.1 (0.0–3.9)	1.1 (0.0–3.4)
Injury	7.9 (3.0–14.8)	7.8 (3.0–14.5)
Skin disease	1.5 (0.0–5.1)	1.5 (0.0–4.8)
Cataract	7.1 (2.4–14.0)	7.2 (2.5–13.5)
Dental	4.4 (0.6–11.7)	4.4 (0.7–10.9)
Kidney stone	6.2 (0.8–16.5)	6.1 (1.1–13.8)
Haemorrhoids	2.7 (0.1–9.6)	2.8 (0.1–7.9)
Urinary tract infection		
Liver disease	12.3 (4.3–24.0)	12.3 (4.5–22.8)
Otitis media	4.5 (0.2–15.3)	4.1 (0.1–13.1)
Tumour	12.1 (2.8–26.9)	12.2 (3.1–25.3)
Typhoid	24.2 (10.0–42.3)	24.1 (10.5–40.7)
Mental disease	7.9 (1.1–20.6)	7.8 (1.1–19.4)
Physical weakness	0.3 (0.0–2.9)	NA
Pneumonia	0.6 (0.0–6.2)	NA
Paralysis	6.5 (0.2–22.1)	
Insomnia	0.5 (0.0–5.6)	0.1 (0.0–0.5)

CrI, credible interval.

Note: Include illness in this table if the minimum sample is greater than 10

## Discussion

To the best of our knowledge, this is the primary attempt in Bangladesh to estimate the cost and economic burden of all illnesses including injuries and chronic illnesses in addition to assessing the inequality in health payment burden in 2011. The study found the mean cost of disease-specific OOP payment was substantially higher in chronic illnesses and injuries than acute illnesses. The study also indicated that high financial burden due to OOP payment was seen in most of the chronic illnesses and injuries, and relatively less in recent acute illnesses. The study also revealed that the disease-specific cost was the highest for paralysis, followed by liver disease, injury, mental disease, tumor, urinary tract infection, heart disease, and cataract. The lowest disease-specific cost was for cold/fever followed by insomnia, physical weakness, and gastritis/peptic ulcer.

Similar to a previous study [21], around 15% of the Bangladeshi households incurred financial catastrophe and 5% of non-poor households became poor due to OOP payments in 2011. Wide variations of economic burden were observed across diseases. Our study found a substantially higher average of OOP payment in broad illness categories in chronic illness and injuries, and comparatively low average OOP payment in recent acute illnesses. Among chronic illnesses, high incidences of catastrophic health expenditure were observed for tumour, liver diseases, heart condition, injuries, and mental illnesses. It was similar with the findings of a study conducted in India where OOP payment was highest for cancer, followed by heart diseases and injuries [22]. In recent acute illnesses, the highest incidence of ruinous payment was typhoid followed by cataract, kidney stone, otitis media, dental, and diarrhea. These results were similar to a study conducted in Nepal, where the OOP payments was considerably higher in chronic diseases and injuries than in acute diseases [12]. Despite the acute nature of typhoid fever, it is associated with a high incidence of catastrophic expenditure due to the disease being more prevalent in lower socioeconomic groups with poor access to adequate sanitation and hygiene. Other contributing factors include a lack of early diagnosis in developing countries and the needs of typhoid fever requiring comprehensive or hospitalized care within a short period of time. This study additionally found that the foremost often reported diseases among study populations were NCDs like cardiovascular disease, rheumatic, arthritis, heart diseases, diabetes, gastritis/peptic ulcer, and asthma, whereas infectious diseases like cold/fever and diarrhea/gastroenteritis.

Although Bangladesh is poised to attain a couple of the Millennium Development Goals (MDG) by 2015, the country needs harder attempt to attain the maximum amount like Sri Lanka since plenty of challenges remain. Problems in health system strengthening, equivalent to improving potency and good governance will require accrued attention within the immediate future; however, the key to further improving equity and accessibility of the system are going to be the reform of health funding. Bangladesh incorporates a dual health care system, with each public and private health services co-existing in most areas. In Bangladesh, about 3.0% of gross-domestic product (GDP) is spent on health in 2015, of which government contribution is merely about a fourth (0.7% of GDP) [23]. The per capita total health expenditure is about US$37per year [23]. Funding of public hospitals comes from revenue; hence, the development budget. Healthcare expenditure per person has inflated slowly over the years with an annual growth rate of 5.9% during 1998–2015 (from $10 in 2000 to $37 by 2015); therefore, the majority of health care funding comes from OOP payments [23]. Two-thirds of total expenditure (67% in 2015) is privately supported through OOP payments, and the remaining one-third comes from the government’s out of tax revenues, development outlays, and international development assistance [23]. Despite the high economic burden for treatment cost of sickness in Bangladesh, there is no national health insurance, neither is the personal insurance market well developed to project population from health care related economic burden [24].

Our study has several strengths. We used robust method to estimate the disease-specific economic burden in Bangladesh. The Bayesian modeling analysis provides not only precise information on the economic burden of specific illnesses, but also gives the conjointly updates on the incidence of catastrophic household health expenditure in Bangladesh by integrating previous information of economic burden in an exceedingly Bayesian framework. This study helps us to understand the magnitude of economic burden as a result of health care cost. However, the analysis protocol and sampling technique in our study were designed to avoid any biases within the results. Even with these strengths, the study encompasses a few limitations. We only examined urban households in one metropolitan area of the country; thus, the results cannot be generalized to the whole country. Yet, the representative nature of the sample implies that the results can be applied to different cities; therefore, the study might mirror the reality of health market participation for an associated large proportion of the Bangladeshi population. Such associate analysis might describe the role of preventable hospital admissions in catastrophic payment. Consumption and expenditure were self-reported leading to a risk of error, although estimates were confirmed completely by different home members or aged persons within the community. Additionally, there is a possibility of recall bias as we used information on health expenditure for each illness episode in the past 30 days. However, such recall period has widely been used in other surveys such as Living Standard Measurement Surveys. We used a Bayesian modeling that enabled us to partly handle the small sample size of some illnesses. Since the informative prior was powerful to induce for every illness, we used a non-informative prior for estimation of the economic burden of each of the sicknesses.

The suggestion from this study is that households with high OOP payments and financial burdens caused by chronic diseases including typhoid, cancer, liver diseases, mental diseases, and heart diseases in Bangladesh were substantial. Households should be protected from the burden of OOP payments through the implementation of the following recommendations:

- After implementing government supported/ public not private health insurance program in LMIC countries, particularly Vietnam, China, and the Philippines, the incidence of catastrophic expenditure substantially declined. The Government of Bangladesh should immediately start health insurance program in its health-financing unit to avoid unpredictable medical expenses.
- Implementation of a subsidy program to a certain extent for diseases with high economic burden like renal diseases, cancer, and heart diseases will alleviate the household from catastrophic expenditure.
- More attention ought to be paid to prevent and control chronic diseases to avoid unpredictable medical expenses.

## Supporting information

S1 Table Self-reported illnesses among household members in Bangladesh, 2011. (DOCX)

## Abbreviations

CrI: Credible interval
CI: Confidence interval
GDP: Gross-domestic product
HIC: High income countries
NCDs: Non-communicable diseases
NGO: Non-governmental organization
OOP: Out-of-pocket
OOPE: Out-of-pocket-Expenses
TK: Taka (Bangladesh local currency)
UHC: Universal Health Coverage
