# Supplementary material for: Disease-specific out-of-pocket healthcare expenditure in urban Bangladesh: A Bayesian analysis
Source: PLoS One. 2020 Jan 14;15(1):e0227565. doi: 10.1371/journal.pone.0227565 (PMC6959568; doi:10.1371/journal.pone.0227565)
Supplement: S1 Table — (DOCX) [file pone.0227565.s001.docx]

Annex Table 1. Self-reported illnesses among household members in Bangladesh, 2011

| Illness | No of household members | Prevalence (95% CI) |
| --- | --- | --- |
| Cold/fever | 1328 | 18.4 (16.6–20.3) |
| Hypertension | 549 | 7.2 (6.2–8.5) |
| Gastritis/peptic ulcer | 392 | 5.4 (4.4–6.5) |
| Rheumatic arthritis | 370 | 5.1 (4.4–5.9) |
| Diabetes | 293 | 3.8 (3.2–4.5) |
| Heart disease | 214 | 2.8 (2.3–3.4) |
| Migraine/headache | 190 | 2.6 (2.2–3.1) |
| Asthma | 154 | 2.0 (1.7–2.4) |
| Diarrhoea/gastroenteritis | 140 | 2.0 (1.5–2.5) |
| Allergy | 96 | 1.3 (1–1.7) |
| Injury | 77 | 1.1 (0.8–1.5) |
| Skin disease | 74 | 1.1 (0.8–1.3) |
| Cataract | 71 | 1.0 (0.7–1.3) |
| Dental | 47 | 0.6 (0.5–0.9) |
| Kidney stone | 33 | 0.5 (0.3–0.7) |
| Haemorrhoids | 39 | 0.5 (0.4–0.8) |
| Urinary tract infection | 32 | 0.5 (0.4–0.7) |
| Liver disease | 41 | 0.5 (0.4–0.7) |
| Otitis media | 24 | 0.4 (0.2–0.5) |
| Tumour | 25 | 0.4 (0.2–0.6) |
| Typhoid | 25 | 0.4 (0.3–0.7) |
| Mental disease | 26 | 0.4 (0.2–0.7) |
| Physical weakness | 25 | 0.3 (0.2–0.5) |
| Pneumonia | 12 | 0.2 (0.1–0.3) |
| Paralysis | 16 | 0.2 (0.1–0.4) |
| Cancer | 8 | 0.1 (0.1–0.3) |
| Food poisoning | 5 | 0.1 (0–0.2) |
| Chicken pox | 4 | 0.1 (0–0.2) |
| Insomnia | 13 | 0.1 (0.1–0.3) |
| Uterine prolapse | 5 | 0.1 (0–0.2) |
| Nasal polyps | 8 | 0.1 (0.1–0.3) |
| Gallbladder stone/cholecystitis | 7 | 0.1 (0.1–0.2) |
| Tuberculosis | 4 | 0.1 (0–0.2) |
| Inguinal hernia | 9 | 0.1 (0.1–0.2) |
| Dengue | 3 | 0.0 (0.0–0.1) |
| Other^b^ | 102 | 1.4 (1.1–1.7) |
| Total | 3300 | 44.9 (42.62–47.25) |

CI, confidence interval.

Note: Red color exclude for mean OOP and cat due to small sample size.
